# Overcoming the Design Challenge in 3D Biomimetic Hybrid Scaffolds for Bone and Osteochondral Regeneration by Factorial Design

**DOI:** 10.3389/fbioe.2020.00743

**Published:** 2020-07-07

**Authors:** Alessandra Dellaquila, Elisabetta Campodoni, Anna Tampieri, Monica Sandri

**Affiliations:** Institute of Science and Technology for Ceramics, National Research Council of Italy (ISTEC-CNR), Faenza, Italy

**Keywords:** factorial design, osteochondral regeneration, hybrid scaffold, biomineralization, collagen cross-linking

## Abstract

Scaffolds for bone regeneration have been engineered by a plethora of manufacturing technologies and biomaterials. However, the performance of these systems is often limited by lack of robustness in the process design, that hampers their scalability to clinical application. In the present study, Design of Experiment (DoE) was used as statistical tool to design the biofabrication of hybrid hydroxyapatite (HA)/collagen scaffolds for bone regeneration and optimize their integration in a multilayer osteochondral device. The scaffolds were synthesized via a multi-step bioinspired process consisting in HA nano-crystals nucleation on the collagen self-assembling fibers and ribose glycation was used as collagen cross-linking method to modulate the mechanical and physical properties. The process design was performed by selecting hydrogel concentration, HA/collagen ratio and cross-linker content as key variables and the fabrication was carried out basing on a full factorial design. Scaffold performances were tested by evaluating porosity, swelling ratio, degradation rate and mechanical behavior as model output responses while physicochemical properties of the constructs were evaluated by TGA, ICP, FT-IR spectroscopy, and XRD analysis. Physicochemical characterizations confirmed the nucleation of a biomimetic inorganic phase and the interaction of the HA and collagenic components. The DoE model revealed a significant interaction between HA content and collagen cross-linking in determining porosity, swelling and mechanical properties of the scaffolds. The combined effect of hydrogel concentration and mineral phase played a key role on porosity and swelling while degradation resulted to be mainly affected by the HA loading and ribose content. The model was then used to determine the suitable input parameters for the synthesis of multi-layer scaffolds with graded mineralization rate, that can be used to mimic the whole cartilage-bone interface. This work proved that experimental design applied to complex biofabrication processes represents an effective and reliable way to design hybrid constructs with standardized and tunable properties for osteochondral tissue engineering.

## Introduction

In the last decades, scaffolds for bone and osteochondral tissue engineering have been studied by using a wide range of materials and manufacturing technologies (Porter et al., 2009; O’Brien, 2011; Hutmacher et al., 2015).

The osteochondral area is a complex multi-layered region composed of articular cartilage and subchondral bone: the articular cartilage is composed of organic and mineralized hybrid layers, separated by an interface called tidemark (Sophia Fox et al., 2009; Simon and Jackson, 2018). The articular extracellular matrix is mainly composed of water (up to 85% of the total weight), collagen and proteoglycans while the tidemark and the calcified cartilage are mineralized regions characterized by a graded increase of the inorganic phase content from 25 to 65 wt. % (Hoemann et al., 2012; Zhang et al., 2012). The inorganic ratio further increases in the bone matrix (65–85 wt. %), where the major mineral phase is a calcium-deficient multi-substituted apatite and collagen is the main organic constituent (Rey et al., 2009; Tampieri et al., 2011).

The bone matrix has been largely replicated *in vitro* by using calcium phosphates (CaPs) as inorganic constituents, mainly hydroxyapatite (HA) and tri-calcium phosphate (TCP), due to their biomimetic properties along with polymeric and hybrid materials (Sabir et al., 2009; Iaquinta et al., 2019; Lei et al., 2019) while scaffolds for cartilage repair are usually made of natural or synthetic polymers (Bhattacharjee et al., 2015; Armiento et al., 2018). The regeneration of the whole osteochondral region is conventionally mimicked by combining these scaffolds into bi or trilayer devices, characterized by inorganic phase gradient, graded mechanical properties and different materials, eventually loaded with growth factors for supporting simultaneous cartilage and bone regeneration (Tampieri et al., 2008; Li et al., 2015; Bittner et al., 2019). Levingstone et al. (2014) fabricated a three-layered osteochondral graft in which the cartilage region was composed of collagen and hyaluronan while the middle and bone layers were produced by adding HA powder to a collagen slurry. In another study, a scaffold for osteochondral regeneration was 3D bioprinted by combining a gradient of nanoHA and poly(lactic-co-glycolic acid) (PLGA) nanospheres incapsulated with chondrogenic transforming growth-factor, demonstrating good osteochondral differentiation *in vitro* (Castro et al., 2015).

The use of biomimetic mineralization methods, consisting of simultaneous and direct nucleation of HA nano-crystals onto self-assembling collagen type I fibrils, represents a solid strategy to closely mimic the chemical, physical and architectural properties of native bone at molecular level (Tampieri et al., 2003; Qiu et al., 2015). The further incorporation of ions, such as magnesium (Mg^2+^), within the HA lattice ensures the nucleation of a highly biomimetic inorganic phase with low crystallinity and enhanced osteoconductive properties (Minardi et al., 2015; Giorgi et al., 2017; Menale et al., 2019).

However, one of the current limitations of hybrid HA/collagen scaffolds is the lack of physiologically relevant mechanical behavior, mainly due to collagen fast degradation and low mechanical stability. A current approach for addressing this drawback is the use of collagen cross-linking methods (Rault et al., 1996; Krishnakumar et al., 2018); among them, non-enzymatic glycation of collagen by ribose enables the stabilization of the collagen matrix while ensuring biocompatibility, low cytotoxicity and non-immunogenic responses (Brodsky et al., 1990; Tampieri et al., 2005; Eleswarapu et al., 2011; Shankar et al., 2017). Ribose has been successfully used for collagen cross-linking for both cartilage and bone tissue engineering. Hybrid mineralized MgHA/collagen scaffolds cross-linked with ribose have shown increased enzymatic resistivity and high citocompatibility (Gostynska et al., 2017; Krishnakumar et al., 2017) and ribose-glycated collagen gels for cartilage regeneration were used to enhance chondrocytes matrix assembly with no cytotoxic effects (Roy et al., 2008).

Despite the significant progresses of tissue engineering in developing new materials and technologies, the clinical translation is still hampered by a lack of standardization in biomedical research. The design and production of biomaterials for regenerative medicine still remains a challenging costly and time-consuming process because of complex native tissues architecture and laborious implementation, that hinder the scale-up toward their clinical application (Holzapfel et al., 2013; Sadtler et al., 2016). A precise control and tuning of the process parameters via reliable statistical methods and a clear understanding of their role on scaffold properties is essential to have robust and scalable devices (Ratner, 2013; Chen et al., 2020). Compared to conventional design approaches, such as *one-variable-at-a-time* and *trial and error* methods, design of experiment (DoE) represents a useful tool to model complex systems as it allows researchers to reduce experiment costs, time and variability by enabling complete data analysis, optimization and outcome prediction (Mason et al., 2003; Leardi, 2009; Dean et al., 2017; Montgomery, 2017).

In the present study, ribose cross-linked magnesium doped HA/collagen scaffolds were synthesized and characterized by TGA, IPC, FT-IR and their performances were systematically investigated by full factorial DoE design. Scaffold porosity, swelling, degradation and mechanical properties in simulated physiological conditions were studied as output responses to the variation of the synthesis parameters. The experimental data were used to build a statistical model of the fabrication process and to optimize the input parameters to mimic different mineralized tissue layers of the osteochondral site.

## Materials and Methods

### Chemicals

HA/collagen scaffolds were synthesized by using type I collagen from equine tendon (Opocrin Spa, Italy) as polymeric phase. Phosphoric acid (H_3_PO_4_, purity 85 wt. %), calcium hydroxide [Ca(OH)_2_, purity 95 wt. %], magnesium chloride hexahydrate (MgCl_2_⋅6H_2_O, purity 99 wt. %), ribose (C_5_H_10_O_5_, purity 99 wt. %), ethanol (C2H6O, purity 95wt. %) and sodium azide (NaN_3_, purity 99%) were obtained from Sigma Aldrich (United States). Phosphate buffer saline (PBS, pH 7.4) was purchased from EuroClone (Italy).

### Factorial Design

Scaffolds were produced following a 2^3^ full factorial design, which provides for the analysis of three independent factors, each of them at two levels, respectively a low and a high level, and requires a number of trials to be performed equal to 2^3^ = 8 at full resolution, thus eight different scaffold formulations (Table 1; Montgomery and Runger, 2003). The model enables the study of linear influence of each main factor on scaffold properties and the analysis of factors interaction. Preliminary studies were performed to choose the factor levels and to standardize the synthesis protocol, reducing the sources of variation (data not shown).

**TABLE 1:** List of experimental input factors (left), with the selected levels (two per each factor, coded as −1 and 1) and the output responses, and (right) the 2^3^ design matrix with the eight scaffold formulations.

**Input factor**	**Model symbol**	**Factor symbol**	**Level**		**Formulation**			
			**−1**	**1**		***X*_1_**	***X*_2_**	***X*_3_**
					F_1_	−1	−1	−1
Filtration time (min)	*X*_1_	t	0.25	5	F_2_	+1	−1	−1
Mineralization rate (%%)	*X*_2_	HA%	30	70	F_3_	−1	+1	−1
Ribose concentration (mM)	*X*_3_	Rib	0	100	F_4_	+1	+1	−1
Output Response					F_5_	−1	−1	+1
1. Porosity (%)					F_6_	+1	−1	+1
2. Swelling Ratio					F_7_	−1	+1	+1
3. Degradation rate (%)					F_8_	+1	+1	+1
4. Compressive Modulus (kPa)								

**FIGURE 1:**
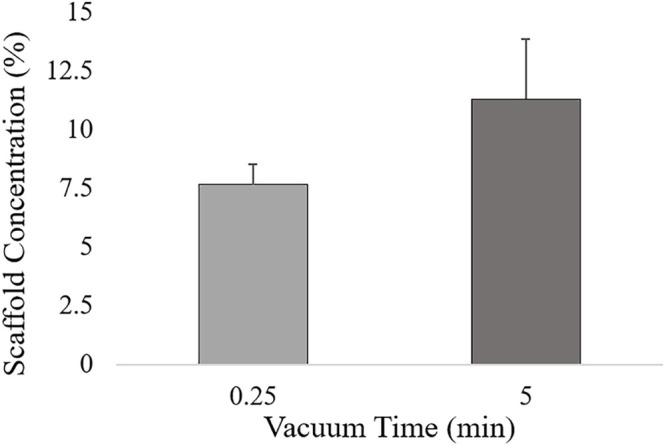
Hydrogel concentration for low (0.25 min) and high (5 min) vacuum time levels, calculated as the ratio between the scaffold weight after freeze-drying and the HA/Collagen wet slurry.

Three main factors were selected: filtration time (t, *X*_1_), mineralization rate (HA%, *X*_2_) and ribose amount (Rib, *X*_3_) (Table 1). The filtration time, defined as the time the HA/collagen slurry was filtered before freeze-drying, determines the hydrogel concentration, measured as the difference between the scaffold weight before and after freeze-drying. From preliminary experiments, a low and a high vacuum time, respectively, equal to 0.25 and 5 min of filtration time were selected, thus ensuring a final scaffold concentration of 7.65 wt. % ± 0.86 wt. % and 11.28 wt. % ± 2.57wt. %, respectively (Figure 1). Hydrogel concentration has been shown to strongly influence scaffold density and hence its morphological, physical and mechanical properties (Drury and Mooney, 2003).

Hydroxyapatite content (wt. %) levels were set, respectively, at 30 and 70% compared to the collagen mass fraction. The selected values can be considered as the minimum and maximum theoretical apatite phase contents for the synthesis of hybrid HA/collagen constructs, with a 70% HA content typical of subchondral bone and 30–40% of HA phase typical of tidemark and calcified cartilage (Tampieri et al., 2008).

For ribose cross-linking, ribose concentrations up to 250 mM have been tested (Boonkaew et al., 2014) and low concentrations (30 mM) have been proven to be effective on intermolecular cross-linking of the collagen fibrils (Chiue et al., 2015; Vicens-Zygmunt et al., 2015; Gostynska et al., 2017). In this work, an intermediate ribose concentration of 100 mM has been selected and the two ribose levels were set, respectively, at 0 (no cross-linking) and 100 mM. Other potential processing variables such as freeze-drying conditions and synthesis temperature were kept constant.

The output responses were porosity percentage, swelling ratio, degradation rate at day 30 and compressive modulus as measure of the mechanical behavior (Table 1).

### Synthesis of HA/Collagen Scaffolds

**FIGURE 2:**
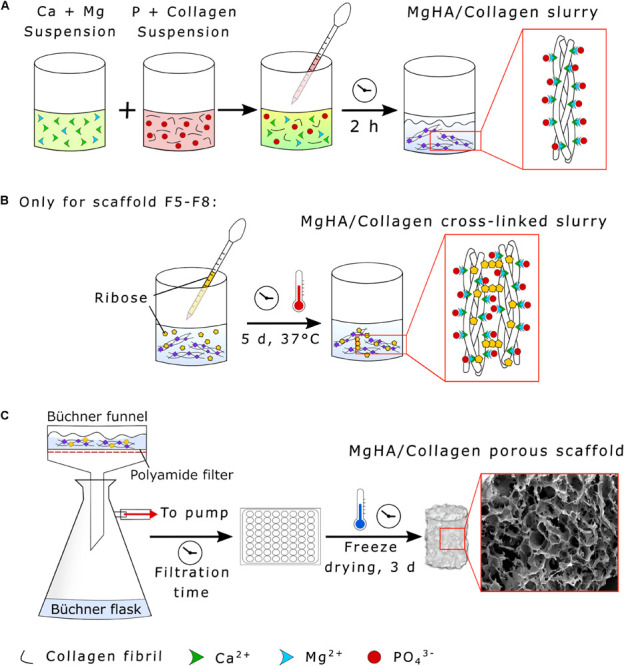
Synthesis of HA/Collagen scaffolds. **(A)** Collagen biomineralization through neutralization process, **(B)** MgHA/Collagen slurry glycation with ribose (only for the scaffolds formulations F5–F8), and **(C)** vacuum filtration and scaffold freeze-drying.

Hybrid HA/collagen scaffolds were prepared through an acid/base reaction (Figure 2). H_3_PO_4_ was dissolved in Milli-Q water and dropped into 100 g of 1 wt. % collagen to prepare an acidic suspension (pH 2.5). A basic suspension (pH 12.0) was obtained by dispersing Ca(OH)_2_ in Milli-Q water followed by mechanical stirring, with subsequent addition of MgCl_2_⋅6H_2_O. The Ca/P (mol) ratio was fixed at 1.67 and the Mg/Ca (mol%) ratio at 5 to achieve a biomimetic Mg-doped HA. The acidic suspension was added to the basic one at room temperature under gentle mechanical stirring, initiating a neutralization process, with a slow decrease of the pH from 12.0 to 7.5 and simultaneous MgHA nano-crystals nucleation onto self-assembling collagen fibers (Figure 2A). After 2 h of HA maturation at room temperature, the slurry was washed with Milli-Q for two consecutive times with a 150 μm sieve to remove the excess of free ions.

Ribose cross-linked scaffolds (Formulations F_5_–F_8_) were prepared by adding 2 g of ribose in a solution of ethanol and PBS (70:30 vol.%) to obtain a final concentration of 100 mM, covering the mineralized slurry and incubating the composite for 5 days at 37°C under constant mechanical shaking (Figure 2B). The cross-linked slurry was then filtered twice by a 150 μm sieve with Milli-Q to eliminate residues.

A Büchner funnel was inserted into a Büchner flask and connected to a vacuum pump. The slurry was then filtered using a polyamide filter for a filtration time of 0.25 or 5 min according to the experimental conditions (see Table 1). The gel was distributed in multiwell plates (48-wells format, JET Biofil^®^, China), covered with an 8 mm diameter polyamide layer and freeze dried (LIO_3000_PLT, 5Pascal, Italy). The process was performed with a controlled freezing temperature slope of −50°C/h up to −40°C and drying temperature of 2°C/hr from -40 to 20°C for 72 h under constant vacuum of 0.1 mbar (Figure 2C).

### Scaffold Characterization

#### Physicochemical Characterization

Before collecting the data to build the DoE model, the physicochemical properties of the hybrid HA/collagen scaffolds were assessed by Thermogravimetric Analysis (TGA), Inductively Coupled Plasma (ICP), Fourier transform infrared spectroscopy (FTIR) and X-Ray Diffraction (XRD).

The actual amount of inorganic phase was calculated by Thermo Gravimetric Analysis (TGA) (STA 449/C Jupiter, Netzsch, Germany). Briefly, 20 mg of the composite were placed in aluminum crucible and pressed to have full contact with the crucible. The experiment was performed in the temperature range of 30-800°C at a heating rate of 10°C/min in air atmosphere. The HA and collagen mass fractions (wt. %) were calculated from the TGA curves and then averaged for the formulations with the same HA/collagen ratio.

The quantitative calculation of Mg^2+^, Ca^2+^, and PO_4_^3–^ ions of the mineral phase was assessed by Inductively coupled plasma-optical emission spectrometry (ICP-OES, Agilent Technologies 5100 ICP-OES, Santa Clara, United States). Briefly, 30 mg of sample were dissolved in 2 ml of nitric acid (65 wt. %) through sonication for 30 min and were then diluted in 100 ml of milli-Q water (the experiment was performed in triplicate for each formulation). 422, 279, and 214 nm were used as wavelengths, respectively, for Ca, Mg and P ions detection.

The chemical interaction between the Mg doped HA and the collagen matrix were studied by Fourier Transform Infrared Spectroscopy (FTIR, Nicolet 380, Thermo Fisher Scientific Inc., Waltham, MA, United States). Samples were prepared by mixing 2 mg of scaffold with 200 mg of anhydrous potassium bromide (KBr) as background element and pressed into 7 mm diameter tablets at 8000 psi. The spectra were acquired in the wavelength range 400–4000 cm^–1^ at 4 cm^–1^ resolution.

X-ray diffraction analyses (XRD, D8 Advance, Bruker AXS, Germany) were performed to investigate the scaffolds crystallinity. To clarify the role of the mineralization time, the presence of ribose and Mg^2+^ ions on samples composition, XRD was performed on scaffolds (i) HA/collagen, 2 h of mineralization, (ii) HA/collagen, 5 days of mineralization, (iii) MgHA/collagen, 5 days of mineralization and (iv) Ribose cross-linked MgHA/collagen (5 days of mineralization). The diffractometer was equipped with a Lynx-eye position-sensitive detector (Cu Kα radiation, α = 1.5418 Å). XRD spectra were recorded at a step size (2θ) of 0.02° from 20° to 80° and a scan speed of 0.5 s.

#### Scaffold Porosity

The theoretical scaffold density *ρ* was calculated as the ratio between mass *m* and volume *V* (cylindrical shape assumption) of the dry scaffold using Equation (1) (*ρ = m/V*). The effective scaffold density ρ_material_ was calculated as the sum of the densities of the scaffold components, i.e., apatite, collagen and water phases assumed equal to 3.16 g/ml (Haverty et al., 2005), 1.45 g/ml (Davidenko et al., 2015) and 1 g/ml respectively and multiplied by the weight percentage within the scaffold. The relative density of the scaffolds was obtained by dividing the theoretical density *ρ* by the ρ_*material*_ and the porosity percentage was calculated from the relative density (Karageorgiou and Kaplan, 2005) following Equation (2):

(1) $$Porosity\left(\%\right)=100x\left(1-\rho /\rho_{m\,a\,t\,e\,r\,i\,a\,l}\right)$$

#### Swelling Ratio and Degradation Rate

The water sorption ability of the scaffolds under physiological conditions was assessed by investigating their swelling behavior. Briefly, cylindrical specimens were weighted in dry conditions and after 24 h of incubation at 37°C in PBS and the swelling ratio was calculated according to Equation (3): [(*W*_*w*_−*W*_*d*_)/*W*_*d*_](Conshohocken, 2011), where W_w_ and W_d_ represent the wet and dry weights respectively.

Degradation rate was calculated as the difference between the initial *W*_*i*_ and the residual mass *W*_*f*_ after 30 days of incubation in PBS solution at 37°C, as described in Equation (4): [100*x*(*W*_*i*_−*W*_*f*_)/*W*_*i*_]. Cylindrical scaffolds were immersed in a PBS solution containing NaN_3_ and maintained under constant shaking; after 30 days of incubation specimens were washed in Milli-Q water and freeze-dried before weighting.

#### Mechanical Characterization

Dynamic mechanical analysis (Q800 DMA, TA Instruments, United States) was performed for the compressive strength calculation (Conshohocken, 2011). Cylindrical samples (diameter of 8 mm and thickness of 10 mm) were soaked in PBS for 24 h before the tests to simulate physiologically wet conditions. Compression tests were performed by setting a ramp force of 0.5 N/min up to 5 N at 37°C.

#### Statistical Analysis

Experimental design and data analysis were performed using Minitab 18 software (Minitab Ltd., United Kingdom). A 2- level full factorial design with default generators, number of factors equal to 3 and 1 block was selected to build the experimental matrix. Sample size of 80 and 10 replicates for each corner point were chosen to ensure a design power of 0.9, with a level of significance α of 0.05 (Bahçecitapar et al., 2016; Montgomery, 2017). Experiments were performed in randomized order according to the design matrix and data are presented as mean ± SD. Experimental data from porosity, swelling ratio, degradation rate and compressive modulus were statistically evaluated by general linear model (GLM) analysis of variance (ANOVA) by selecting interactions through third order. The following regression equation was used to evaluate each output (Equation 5):

$$Y=b_{0}+b_{1}\,X_{1}+b_{2}\,X_{2}+b_{3}\,X_{3}+b_{12}\,X_{1}\,X_{2}+$$

(2) $$b_{13}\,X_{1}\,X_{3}+b_{23}\,X_{2}\,X_{3}+b_{123}\,X_{1}\,X_{2}\,X_{3}$$

where *Y* is the measured response, *b*_0_ represents the intercept, *b*_*i,j,k*_ are the linear coefficients, *b*_*ij*,*ik*,*jk*_ are the estimated coefficients of the two-interaction terms and *b*_*ijk*_ is the three-interaction coefficient. ANOVA results and Pareto charts were evaluated to find the statistical significance of independent variables and interaction terms and eventually reduce the model. Residuals analysis was performed to validate ANOVA assumptions. Main effect, interaction and contour plots were generated to visualize the influence of factors and interactions on the responses.

## Results and Discussion

### Physicochemical Characterization

**FIGURE 3:**
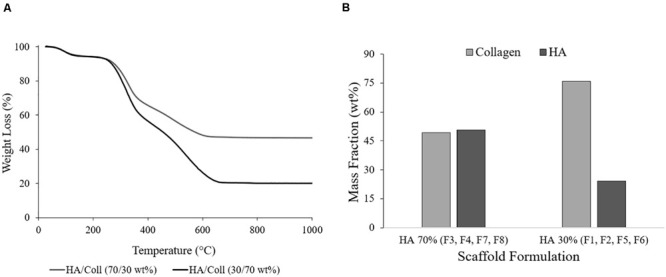
**(A)** Representative TGA curves of the formulations HA/collagen 70/30 wt. % and 30/70 wt. % (F1 and F7) and **(B)** average data of the HA and collagen mass fractions (wt. %).

Results from TGA showed that the actual HA/collagen ratio for the 70% HA theoretical formulations (F_3_, F_4_, F_7_, F_8_) was 50.72/49.28 wt. %, with a final residual weight comparable to the natural bone composition (Figure 3A; Rana et al., 2017). For the 30% HA theoretical formulations (F_1_, F_2_, F_5_, F_6_) the final ratio HA/collagen was 24.17/75.83 wt. % (Figure 3B), showing a mineral content that can be compared to the human articular calcified cartilage (Hoemann et al., 2012).

The ICP data (averaged for the formulations HA 70 and 30%, respectively, Table 2) confirmed the presence of Mg^2+^ ions in the HA reticulum as well as the nucleation of a calcium deficient low crystalline (1.45–1.60) mineral phase for the 30% HA scaffold formulations due to Mg substitution.

**TABLE 2:** ICP average values for the formulations HA 70% and HA 30%.

**Formulation**	**Ca/P (mol)**	**Mg/Ca (mol%)**	**(Ca+Mg)/P (mol)**
HA 70% (F3, F4, F7, F8)	1.74 ± 0.028	3.62 ± 0.45	1.80 ± 0.034
HA 30% (F1, F2, F5, F6)	1.44 ± 0.028	1.38 ± 0.096	1.46 ± 0.029

**FIGURE 4:**
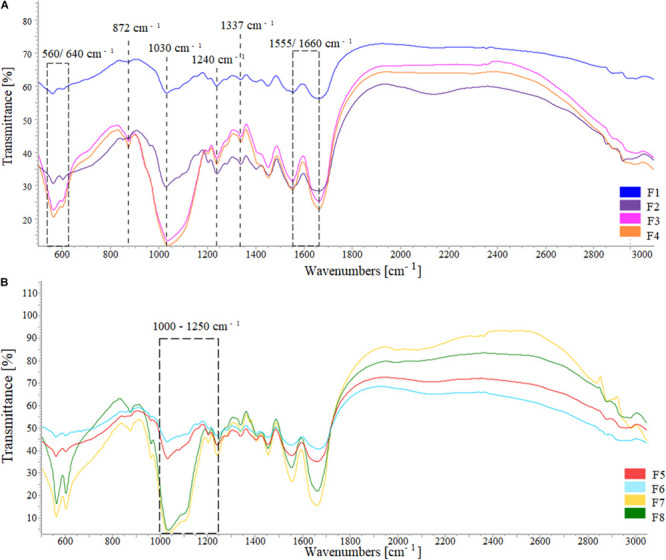
FTIR spectra of **(A)** the formulations HA/collagen and **(B)** the formulations HA/collagen after ribose cross-linking.

FTIR spectra showed the typical HA phosphate group (PO_4_^3–^) bands at 560–640 and 1030 cm^–1^ and the collagen type I amide I, II, and III absorption bands (at 1659, 1555, and 1180–1300 cm^–1^, respectively) (Belbachir et al., 2009), with a broad spectrum typical of a low crystalline composite material (Figure 4). The shift from 1340 to 1337 cm^–1^ can be attributed to the collagen carboxyl groups stretching due to their interaction with the apatite nanocrystals while the band at 872 cm^–1^ indicated the carbonation of MgHA nucleated on the collagen fibrils (Minardi et al., 2015). For the ribose cross-linked scaffolds, typical ribose bands corresponding to C-O, C-C stretching vibrations and the C-OH and C-C-O bending vibrations were found in the 1000–1200 cm^–1^ range (Roy et al., 2010; Huang et al., 2016).

**FIGURE 5:**
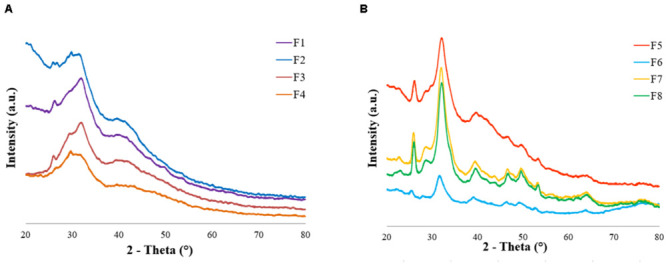
XRD patterns of **(A)** formulations F1–F4 (2 h of HA maturation time, no ribose cross-linking) and **(B)** formulations F5–F8 (5 days of HA maturation time, ribose cross-linking).

The XRD data showed the presence of the main HA peaks at 25° and 32° according to the main lattice reflections of the JCPDS-ICDD file (Card # 09-0432). The pattern exhibited a scarcely crystalline profile of the mineral phase, with a broad profile, in good agreement with FTIR results, confirming the capability of the adopted biomineralization protocol to closely mimic the natural bone features (Figure 5 and Supplementary Figure S1; Stock, 2015; Ding et al., 2019). The scaffold formulations without ribose (F_1_ to F_4_) were nucleated for 2 h before washing and freeze-drying while the formulations containing ribose (F_5_ to F_8_) were incubated for 5 days to cross-link the collagen. These two different experimental settings determined different HA maturation time (2 h versus 5 days) (Figures 5A,B), as assessed by the XRD pattern: the formulations containing ribose showed a higher crystallinity due to the longer HA maturation time. XRD analysis was performed also on scaffolds incubated for 5 days w/o magnesium and w/o ribose in order to assess the influence of foreign ions and sugars on the inorganic phase crystallinity. The presence of Mg^2+^ and ribose contribute in lowering the HA crystallinity but with no significant variation, as previously demonstrated (Supplementary Figure S1; Minardi et al., 2015).

### Factorial Design Output Responses

**FIGURE 6:**
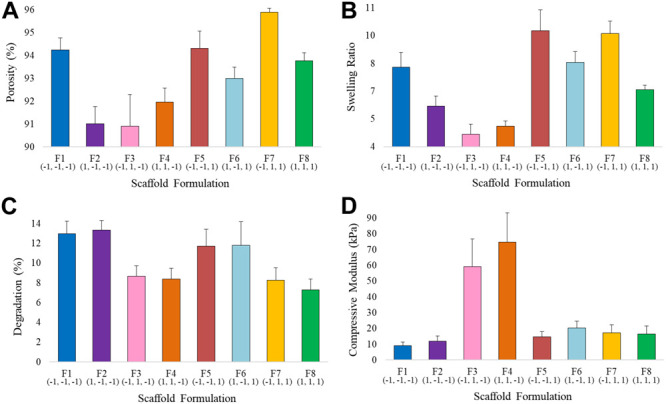
Average values of **(A)** Porosity, **(B)** Swelling Ratio, **(C)** Degradation Rate, and **(D)** Compressive Modulus for the eight scaffold formulations.

Average values (*n = 10*) of the porosity percentage, swelling ratio, degradation rate and compressive modulus for each scaffold formulation are shown in Figure 6 and Supplementary Table S1. The variation of the input variables, i.e., filtration time (X_1_), mineralization rate (X_2_) and ribose content (X_3_), resulted in a significant change of the responses. All the scaffold formulations showed a high porosity percentage, from 90 to 96%, which ensures a high surface area suitable for *in vitro* cells growth, nutrients diffusion and waste removal and fundamental in tissue engineering devices (Freed et al., 1994; Puppi et al., 2010). Water-binding ability, expressed as swelling ratio, varies from 4.18 ± 0.54 for F_3_ (0.25 min, 70% of HA, 0 mM of ribose) to 9.77 ± 1.11 for F_5_ (0.25 min, 30% HA, 100 mM), showing minimum values for 70% HA non-crossliked scaffolds. Degradation rate, calculated as the weight loss after 30 days of incubation in PBS, ranges from about 7 to 12% and resulted to be lower for scaffolds with 70% of apatite content (F_3_, F_4_, F_7_, and F_8_). The compressive modulus reaches values up to 75 kPa in scaffolds with high level of HA% (F_3_ and F_4_).

### Data Analysis From Factorial Design

Data from ANOVA and Pareto charts (Supplementary Table S2 and Supplementary Figure S2) were used to identify the significant factors and to find the regression coefficients based on p-value evaluation. The non-significant terms (*p*-value < α, with level of significance α of 0.05) could be deleted and the model reduced just for the degradation rate, in which the interaction terms resulted to be non statistically significant. For the other outputs, the non significant terms were included to respect the model hierarchy, leading to the following regression equations:

$$P\,o\,r=97.10-1.36\,X_{1}-0.089\,X_{2}-0.04\,X_{3}+0.023\,X_{1}\,X_{2}+$$

(3) $$0.012\,X_{1}\,X_{3}+0.0013\,X_{2}\,X_{3}-0.00027\,X_{1}\,X_{2}\,X_{3}$$

$$S\,w=10.72-0.84\,X_{1}-0.094\,X_{2}-0.008\,X_{3}+0.013\,X_{1}\,X_{2}+$$

(4) $$0.0069\,X_{1}\,X_{3}+0.00092\,X_{2}\,X_{3}-0.0002\,X_{1}\,X_{2}\,X_{3}$$

(5) $$\mathrm{Degr}=16.24-0.108\,X_{2}-0.011\,X_{3}\,\left(\mathrm{ReducedModel}\right)$$

$$E=-28.17-1.38\,X_{1}+1.24\,X_{2}+0.4\,X_{3}+0.067\,X_{1}\,X_{2}+$$

(6) $$0.036X_{1}X_{3}+-0.012X_{2}X_{3}-0.001X_{1}X_{2}X_{3}$$

The analysis of residual plots was carried out for each output: the normal probability plots and the histograms of residuals confirmed a normal distribution of data with no skewness and few outliers for porosity, swelling ratio and degradation rate (Supplementary Figure S3). The normal probability plot for compressive modulus showed a p-value lower than α and a curved distribution, violating the assumption of normal distribution and indicating that a second order term needs probably to be included in the model. However, considering the results from ANOVA analysis, the model was considered sufficiently adequate to describe the dataset. High values of the coefficient of determination *R*^2^, adjusted *R*^2^ (*R*^2^*_*adj*_*) and predicted *R*^2^ (*R*^2^_Pred_) (close to 1) were obtained for all the outputs, indicating a good fit of the response equations with the experimental data and showing a good predictive capability of the model (Supplementary Table S2). The “Residuals versus fits” and “Residual versus order” graphs confirmed the random data distribution and the residuals independency for all the responses.

Interaction and contour plots of porosity, swelling and compressive modulus were evaluated to investigate the influence of factor interactions on scaffold performances. The main effect plots of degradation have been studied since there was no evidence of interaction among factors for this response. The main effect plots for the other responses are shown in Supplementary Figure S4.

#### Porosity

**FIGURE 7:**
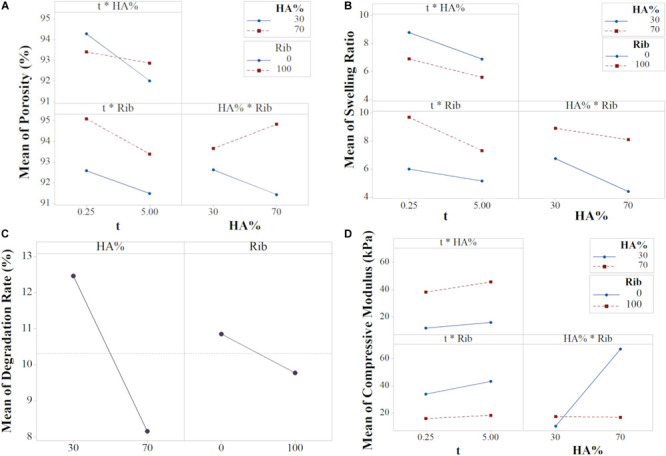
Interaction plots for **(A)** Porosity, **(B)** Swelling Ratio, **(D)** Compressive Modulus, and **(C)** Main plot for Degradation (data from the reduced model).

The input variables X_1_ and X_3_ had the largest effect (Equation 6 and Supplementary Figure S2A), meaning they are the most influencing parameters in determining the scaffold porosity (Supplementary Figure S4). However, basing on the ANOVA results, the interactions t^∗^HA% and HA%^∗^Rib resulted to be statistically significant, thus the interaction effects needed to be interpreted since the main effects *per se* can be misleading. The interaction plot (Figure 7A) reports the mean of porosity values as function of the combination of the three input variables X_1,_ X_2_ and X_3_. The lack of parallelism indicates that, when tuning one of the three factors, the output variation is dependent on the other factors (Montgomery and Runger, 2003).

Although the HA% resulted to be not statistically significant, as shown in the main effects plot (Supplementary Figure S4A), the interaction effect between porosity and filtration time is dependent on the mineral phase content. The t^∗^HA% graph shows that shorter filtration times leads to higher porosity, with similar values for both the HA% level tested. A filtration time of 5 min decreases significantly the porosity in 30% HA scaffolds while the output remains almost constant for 70% HA formulations. As previously shown, scaffold porosity is inversely proportional to stiffness (Sudarmadji et al., 2011) so porosity in 70% HA formulation can be expected to be lower for any input parameter tested. However, the results from both the main and interaction effects show a high impact of the filtration time on the final porosity, with 5 min filtration time inducing a drastic porosity decrease for 30% HA content. The behavior can be attributed to the capability of the constructs to be further compressed when the HA% is lower. A high vacuum time thus eliminates more water in 30% HA slurries, leading to higher scaffold concentrations and consequently to lower porosity percentage (Figure 1) while in 70% HA scaffolds formulation the porosity remains almost constant for both the filtration time tested because of the high mineral phase content, that reduces the scaffold compressibility. The input values t of 0.25 min and HA 30% resulted in the highest scaffold porosity.

The HA%^∗^Rib graph showed that the presence of ribose is responsible for higher porosity percentages for both the HA% tested. While a small difference of the output is noticed in 30% HA formulations, either cross-linked or not, in 70% HA scaffolds the ribose substantially contributes in increasing the porosity compared to non cross-linked matrices. The trend can be related to the porogen effect of ribose, that increases both the pore size and porosity degree, as previously reported (Karageorgiou and Kaplan, 2005) and is confirmed by the X_3_ main effect plot (Supplementary Figure S4A). In absence of ribose, 70% HA scaffolds resulted less porous than 30% HA, probably due to the higher mineral content, as discussed above.

#### Swelling Ratio

The swelling ratio equation showed that all the interaction terms are statistically significant. Both the plots t^∗^Rib and HA%^∗^Rib showed that the effect of Rib on swelling is greater for 100 mM compared to 0 mM. For the t^∗^HA%, the 30% HA content determines higher swelling in comparison to higher HA/collagen ratio (Figure 7B).

The X_1_X_3_ interaction shows that, for a slurry filtered with low filtration time, the presence of ribose plays a key role in determining higher swelling ratio compared to non cross-linked scaffolds. For prolonged filtration time, the swelling ratio resulted to be reduced, with little effect of ribose. However, the highest decrease is noticed in cross-linked scaffolds due to an increased collagen stability at higher hydrogel concentration (Hiremath and Vishalakshi, 2012).

Conversely, the effect of ribose on swelling resulted reversed when the influence of HA% is considered (X_2_X_3_ interaction): ribose has a large effect on average swelling in 70% scaffolds (formulations F_3_ and F_4_), showing that the absence of cross-linking leads to a drastic reduction of water binding capability. In contrast to the majority of the cross-linking agents, that reduce the collagen swelling capability (Reháková et al., 1996; Charulatha and Rajaram, 2003), the bonding between the aldehyde groups of ribose with the amino groups of collagen during cross-linking influences the swelling positively (Roy et al., 2010; Krishnakumar et al., 2017). The effect is more pronounced for 30% HA scaffolds due to the higher collagen availability and can be related to the porogen action that the ribose exerts, leading to an increased water uptake capability compared to non cross-linked matrices (Figure 7A). Thus, the use of non-enzymatic glycation ensures the fabrication of stiffer collagenic scaffolds while ensuring high surface/volume ratios, able to promote cells adhesion and matrix colonization (Gostynska et al., 2017; Krishnakumar et al., 2018). The analysis of X_1_X_2_ interaction showed that the HA% content has a higher effect on swelling for low filtration time and the maximum swelling ration is obtained for 30% HA scaffolds filtered for 0.25 min.

#### Degradation Rate

The regression equation of degradation rate revealed the statistical significance of the two main effects HA% and ribose while no significant interaction effects were found for the analyzed dataset (Supplementary Figure S2). These results enabled the model to be reduced, thus the interaction terms and the variable X_1_ could be eliminated in order to have more precise predictions, as condirmed by the values of *R*^2^_adj_ and *R*^2^_peed_ of the reduced model (Supplementary Table S2). Figure 7C shows the main effect plot for the relevant input factors, with each graph reporting the mean response for the two levels studied. The average degradation resulted to be 12.47 and 8.16% for 30 and 70% HA scaffold formulations, respectively. In fact, a higher mineral content content has been shown to affect the degradation degree of the polymeric phase by stabilizing the scaffold structure, a mechanism that results of great interest for *in vivo* applications, when the scaffold degradation kinetics needs to be properly tuned to match the tissue regeneration timing (Navarro et al., 2006; Rezwan et al., 2006; Tampieri et al., 2008).

With a similar trend, the presence of cross-linker reduced the mean degradation rate from 10.85 to 9.78% in comparison to non-crosslinked scaffolds. Ribose is responsible for collagen glycation through a protein-to-protein cross-linking mechanism, that leads to a reduced scaffold solubility (Krishnakumar et al., 2017).

#### Mechanical Properties

The compressive modulus equation showed that the X_2_X_3_ interaction is statistically significant. The HA%^∗^Rib interaction plot shows that in the 30% HA formulation the effect of ribose is lower compared to the 70% HA, however it can be noticed that the glycation process with a ribose concentration of 100 mM increases the modulus of about 1.6-fold (F_2_ and F_6_ compared to F_1_ and F_5_ respectively). 1 wt. % collagen gels have been reported to have an elastic modulus of 4kPa and ribose at concentration of 250 mM is responsible for increasing their stiffness up to 10 times (Sheu et al., 2001; Roy et al., 2010; Mason et al., 2013). In our study, the effect of the ribose on collagen stiffness is lower compared to literature data since the efficiency of the glycation reaction can be hampered by the presence of the mineral phase, that reduces the availability of collagen free sites. Still, the modulus resulted to be higher in 30% HA cross-linked scaffolds compared to absence of glycation. The prevailing interaction of HA% with the collagen fibrils during the biomineralization process is further confirmed in 70% HA formulation, for whom the modulus remained almost constant in cross-linked devices (F_5_–F_8_) while the specimens with non cross-linked collagen matrix resulted to be almost four times stiffer (average compressive modulus higher than 70 kPa), as confirmed by the main effects plot (formulations F_3_ and F_4_, Figures 6D, 7D and Supplementary Figure S3C). The result can be related to the HA nucleation process: in scaffolds with 70% HA, collagen shows a reduced number of free sites for the ribose bond since it is already linked to apatite nano-crystals. This condition determines heterogeneities and reduction of the cross-linking (Anseth et al., 1996), thus inducing a non-significant increase of mechanical strength in 70% HA cross-linked scaffolds.

Collagen cross-linking has shown to affect both the compressive modulus and the swelling in 30% HA matrices, producing an effect that can be compared to the function of proteoglycans (PGs) in the deep zone of the cartilage site. In fact, the negative charge of PGs determines the tissue swelling pressure, making these molecules acting as cartilage bio-bearings (Hussainova and Ghaemi, 2008; Dëdinaitë, 2012; Jahn et al., 2016; Armiento et al., 2018). Ribose cross-linking has shown to induce higher fluid uptake properties in 30% HA devices: the cross-linking caused an increase of the average swelling from 6.75 to 8.92% compared to non-cross-linked scaffolds (F_5_, F_6_ versus F_1_, F_2_) (Responte et al., 2007; Roy et al., 2008). In a similar fashion, the stiffness of the matrices increases from 10.55 kPa to 17.48 kPa, making the glycation process a valid approach for mimicking the articular cartilage matrix behavior *in vivo* (Gostynska et al., 2017).

**FIGURE 8:**
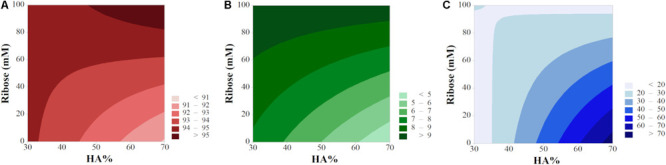
Contour plots for **(A)** Porosity, **(B)** Swelling Ratio, and **(C)** Compressive Modulus.

2D contour maps helped in further elucidating the input/output relationship and have been plotted as function of X_2_ and X_3_ (vacuum time X_1_ was maintained at a fix level). In order to show the optimum of each response, porosity and swelling have been plotted by keeping the filtration time *t* = 0.25 min while the high level (*t* = 5 min) has been selected for degradation and compressive modulus (Figure 8). The lack of parallelism in isporesponse lines indicated the presence of relevant interactions (Leardi, 2009), except for the degradation rate, in agreement with the model equation (Supplementary Figure S5). High levels of both HA% and ribose ensure the maximum porosity rate (>95%, Figure 8A). Swelling behavior increased for cross-linked scaffolds while resulted to be minimum for high HA% level in non cross-linked devices. As confirmed by interaction plots, the 70% HA formulation provided the maximum compressive modulus (>70 kPa) in absence of ribose.

### Response Optimization for the Design of Osteochondral Multi-Layer Scaffolds

Starting from the DoE model built on our experimental data, response optimization was performed to set the ideal input parameters for the synthesis of multi-layer scaffolds with graded HA content that can mimic the osteochondral site.

Constructs for clinical application should provide suitable mechanical support to the injured area as well as degradation kinetics comparable to the tissue regeneration (Hutmacher, 2000; Pivonka and Dunstan, 2012). Therefore, in the optimization process, the mimicry of the subchondral bone was ensured by maximizing the compressive modulus while minimizing the degradation rate and the porosity percentage. Since the degradation kinetics is accelerated *in vivo* compared to *in vitro* studies (Lam et al., 2009), a minimum degradation rate was chosen during the optimization. A minimization of the porosity was selected because the subcondral bone is corticalized, showing a reduced porosity compared to the adjacent tissues (Burr, 2004). However, a porosity percentage exceeding 90% was secured, as required by tissue engineering criteria (Puppi et al., 2010). Maximum swelling ratio and porosity percentage, with a constrained HA% value ranging from 30 to 40% were chosen as optimal input variables for the calcified cartilage region.

Results from the simulation showed that calcified cartilage can be mimicked in this experimental setup by using low level of filtration time, 40% of HA content and a ribose concentration of 100 mM while the F_4_ formulation represents the optimal solution to mimic subchondral bone (5 min of vacuum time, 70% HA and no ribose) (Table 3). Composite desirability (D) values revealed a good optimization of the whole set of responses for both cartilage (*D* = 0.8) and bone (*D* = 0.63). Furthermore, low values of Standard Error Fit (SE Fit) and narrow ranges of 95% confidence interval (CI) and prediction interval (PI) indicate a precise estimate of the mean responses and prediction accuracy.

**TABLE 3:** Optimization values and simulation solutions (estimated input, output fit and statistical intervals) for subchondral bone and calcified cartilage, where ↓ = min, ↑ = max, D = Composite Desirability (0–1), CI = Confidence Interval, PI = Prediction Interval.

	**Optimization**		**Solution**					
	**Output**	**Goal**	**Estimated Input**		**Output *Fit***		**95% CI**	**95% PI**
**Subchondral bone *D* = 0.628**								
	Por	↓	t (min)	5	Por (%)	91.96	(91.50; 92.41)	(90.45; 93.46)
	Degr	↓	HA (wt. %)	70	Degr (%)	8.41	(7.51; 9.30)	(5.44; 11.37)
	E	↑	Rib (mM)	0	E (kPa)	74.86	(68.78; 80.94)	(54.69; 95.04)
**Articular cartilage *D* = 0.804**								
	Por	↑	t (min)	0.25	Por (%)	94.71	(94.35; 95.07)	(93.23; 96.18)
	Sw	↑	HA (wt. %)	40	Sw	9.73	(9.41; 10.06)	(8.39; 11.07)
			Rib (mM)	100				

## Conclusion

In this work, experimental design (DoE) was used to investigate the role and interaction of critical process parameters on hybrid HA/collagen scaffold performances. A 2^3^ factorial design was chosen to study the role of hydrogel concentration (modulated by varying the filtration time), hydroxyapatite content and ribose glycation on mineralized constructs in order to build a model for the fabrication of multilayer scaffolds for osteochondral regeneration. The physicochemical analyses confirmed the nucleation of a poorly crystalline HA mineral phase, the incorporation of Mg^2+^ ions in the HA lattice and ribose glycation for cross-linked scaffolds. XRD data confirmed that HA crystallinity is always low due to its growth in close interaction with collagen and mainly affected by the maturation time. Data collected for scaffold porosity, swelling, degradation rate and compressive modulus were investigated as outputs and the values obtained from the ANOVA confirmed a good predictability of the mathematical models.

Results revealed that the degradation rate was negatively affected by high levels of HA% and ribose as individual factors since a higher mineralization degree, as in sample 70% HA, and cross-linking are responsible to stabilize the scaffold structure. The combined use of low filtration time and low HA% produced scaffolds showing the maximum degree of porosity and swelling while the interaction between HA% and ribose resulted to be the most significant factor in determining the scaffolds porosity, swelling and mechanical behavior. Particularly, the glycation of collagen led to an increase of both porosity and swelling degrees, showing that ribose acts on collagen matrices as a porogen agent and augments the water uptake capability. The effect of ribose on stabilizing the collagen structure was also observed in 30% HA cross-linked formulations, that showed an increased compressive modulus. The results confirmed the advantage of using non-enzymatic glycation of collagen matrices as non-toxic and biocompatible method to obtain highly porous scaffolds with enhanced mechanical stability. Furthermore, ribose glycation caused a response comparable to that of cartilage proteoglycans in the mineralized cartilage, thus ensuring a proper swelling pressure for resisting compressive loads.

The model was then used to optimize the input variables for the synthesis of osteochondral devices: results showed that the ideal input parameters to fabricate calcified cartilage and subchondral bone could be designed in a fast and easy way from the DoE model and the experimental data previously collected.

This study demonstrated that factorial design is an effective statistical method for analyzing, optimizing and standardizing complex biomimetic processes and more sophisticated DoE models for the analysis of the whole osteochondral region, including non-mineralized cartilage, are currently under investigation. We have previously demonstrated the cytocompatibility of our constructs for cartilage and bone regeneration (Gostynska et al., 2017; Krishnakumar et al., 2017) and further biological evaluation will be pursued in future studies.

## Data Availability Statement

All datasets presented in this study are included in the article/Supplementary Material.

## Author Contributions

AD, EC, and MS conceived the presented study. AD carried out the experiment with support from EC and MS in the planning and analysis of data. MS and AT supervised the project. AD wrote the manuscript with input from all authors. All authors contributed to the article and approved the submitted version.

## Conflict of Interest

The authors declare that the research was conducted in the absence of any commercial or financial relationships that could be construed as a potential conflict of interest.
