# Supplementary material for: Overcoming the Design Challenge in 3D Biomimetic Hybrid Scaffolds for Bone and Osteochondral Regeneration by Factorial Design
Source: Front Bioeng Biotechnol. 2020 Jul 7;8:743. doi: 10.3389/fbioe.2020.00743 (PMC7381347; doi:10.3389/fbioe.2020.00743)
Supplement: Supplementary file 1 [file Table_1.DOCX]

***Supplementary Material***

**Supplementary Figures and Tables**

Table S1. List of experimental output results (mean values) for the eight scaffold formulations.

| Formulation | | t | HA% | Rib | Porosity (%) | Swelling  Ratio | Degradation (%) | Compressive Modulus (kPa) |
| --- | --- | --- | --- | --- | --- | --- | --- | --- |
| F_1_ | -1 | -1 | -1 | 94.25 ± 0.514 | 7.80 ± 0.804 | 12.99 ± 1.225 | 9.05 ± 2.267 |  |
| F_2_ | +1 | -1 | -1 | 91.01 ± 0.764 | 5.69 ± 0.548 | 13.35 ± 0.962 | 12.05 ± 3.100 |  |
| F_3_ | -1 | +1 | -1 | 90.91 ± 1.384 | 4.18 ± 0.540 | 8.66 ± 1.050 | 59.14 ± 17.646 |  |
| F_4_ | +1 | +1 | -1 | 91.96 ± 0.629 | 4.60 ± 0.285 | 8.41 ± 1.091 | 74.86 ± 18.437 |  |
| F_5_ | -1 | -1 | +1 | 94.31 ± 0.757 | 9.77 ± 1.114 | 11.72 ± 1.685 | 14.66 ± 3.323 |  |
| F_6_ | +1 | -1 | +1 | 93.01 ± 0.486 | 8.06 ± 0.567 | 11.80 ± 2.390 | 20.31 ± 4.269 |  |
| F_7_ | -1 | +1 | +1 | 95.89 ± 0.184 | 9.62 ± 0.664 | 8.27 ± 1.276 | 17.39 ± 4.862 |  |
| F_8_ | +1 | +1 | +1 | 93.78 ± 0.351 | 6.58 ± 0.248 | 7.31 ± 1.081 | 16.52 ± 5.091 |  |

Table S2. Summary of Analysis of Variance (ANOVA) and Model Summary for each output.

| Source | Degree of Freedom (DF) | Seq Sum of squares (SS) | Contribution | Adj Mean square (MS) | F-value | P-value |
| --- | --- | --- | --- | --- | --- | --- |
| *Porosity* |  |  |  |  |  |  |
| Model | 7 | 215.11 | 85.27 % | 30.74 | 59.52 | 0.000 |
| Linear | 3 | 137.47 | 54.49 % | 45.86 | 88.75 | 0.000 |
| 2-Way Interactions | 3 | 45.17 | 17.90 % | 15.06 | 29.16 | 0.000 |
| 3-Way Interactions | 1 | 32.47 | 12.87 % | 32.47 | 62.90 | 0.000 |
| Error | 72 | 37.17 | 14.73 % | 0.52 |  |  |
| Total | 79 | 252.29 | 100.00 % |  |  |  |
| Model Summary | S= 0.719 | $R^{2}$= 0.853 | $R_{adj}^{2}$=0.838 | $R_{pred}^{2}$=0.813 |  |  |
| *Swelling* |  |  |  |  |  |  |
| Model | 7 | 318.12 | 91.26 % | 45.45 | 107.34 | 0.000 |
| Linear | 3 | 274.29 | 78.68 % | 91.43 | 215.95 | 0.000 |
| 2-Way Interactions | 3 | 25.27 | 7.25 % | 8.43 | 19.90 | 0.000 |
| 3-Way Interactions | 1 | 18.56 | 5.32 % | 18.56 | 43.83 | 0.000 |
| Error | 72 | 30.48 | 8.74 % | 0.42 |  |  |
| Total | 79 | 348.60 | 100 % |  |  |  |
| Model Summary | S= 0.651 | $R^{2}$= 0.913 | $R_{adj}^{2}$=0.904 | $R_{pred}^{2}$=0.892 |  |  |
| *Degradation Rate (Full Model)* | | | | | | |
| Model | 7 | 402.31 | 73.55 % | 57.47 | 28.60 | 0.000 |
| Linear | 3 | 395.27 | 72.26 % | 131.76 | 75.57 | 0.000 |
| 2-Way Interactions | 3 | 6.81 | 1.25 % | 2.27 | 1.13 | 0.343 |
| 3-Way Interactions | 1 | 0.23 | 0.04 % | 0.23 | 0.11 | 0.737 |
| Error | 72 | 144.67 | 26.45 % | 2.01 |  |  |
| Total | 79 | 546.91 | 100.00 % |  |  |  |
| Model Summary | S= 1.418 | $R^{2}$= 0.736 | $R_{adj}^{2}$=0.709 | $R_{pred}^{2}$=0.674 |  |  |
| *Degradation rate (Reduced Model)* | | | | | | |
| Model | 2 | 394.53 | 72.13 % | 197.26 | 99.63 | 0.000 |
| Linear | 2 | 394.53 | 72.13 % | 197.26 | 99.63 | 0.000 |
| Error | 77 | 152.46 | 27.87 % | 1.98 |  |  |
| Lack-of-Fit | 5 | 7.78 | 1.42 % | 1.56 | 0.77 | 0.57 |
| Pure Error | 72 | 144.67 | 26.45 % | 2.01 |  |  |
| Total | 79 | 546.98 | 100 % |  |  |  |
| Model Summary | S= 1.407 | $R^{2}$= 0.721 | $R_{adj}^{2}$=0.714 | $R_{pred}^{2}$=0.699 |  |  |
| *Compressive Modulus* | | | | | | |
| Model | 7 | 42603.7 | 86.40 % | 6086.20 | 65.37 | 0.000 |
| Linear | 3 | 25620.3 | 51.96 % | 8540.10 | 91.72 | 0.000 |
| 2-Way Interactions | 3 | 16520.6 | 33.51 % | 5506.90 | 59.14 | 0.000 |
| 3-Way Interactions | 1 | 462.8 | 0.94 % | 462.80 | 4.97 | 0.029 |
| Error | 72 | 6704.0 | 13.60 % | 93.10 |  |  |
| Total | 79 | 49307.7 | 100 % |  |  |  |
| Model Summary | S= 9.649 | $R^{2}$= 0.864 | $R_{adj}^{2}$=0.851 | $R_{pred}^{2}$=0.832 |  |  |

| **(A)** | **(B)** | **(C)** |
| --- | --- | --- |
| **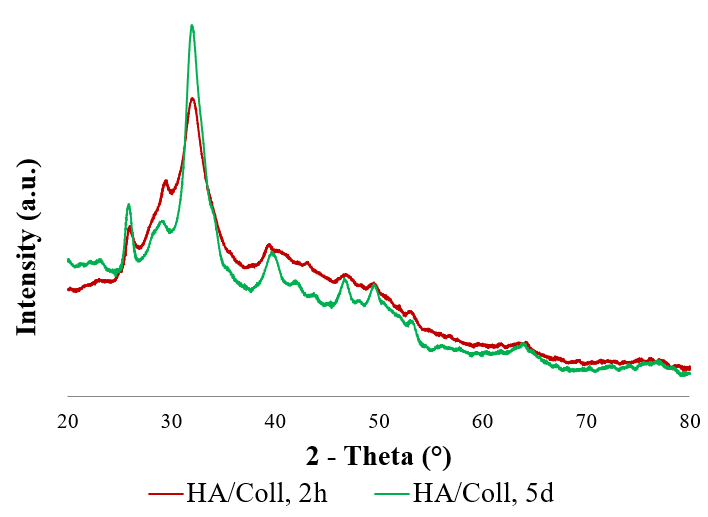** | **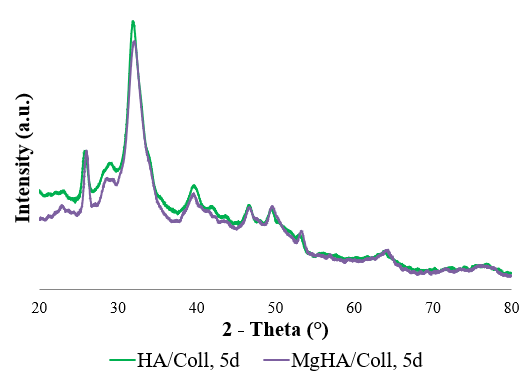** | **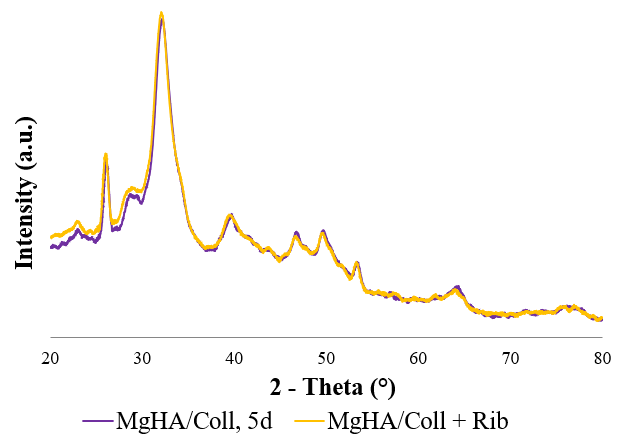** |

**Figure S1.** XRD Patterns of the mineralized collagen scaffolds. Comparison of inorganic phase crystallinity for scaffolds **(C)** HA/collagen aged for 2h or 5d, **(D)** w/o magnesium (5d of HA maturation) and **(E)** w/o ribose (5d of HA maturation).

| **(A)** | **(B)** |
| --- | --- |
| **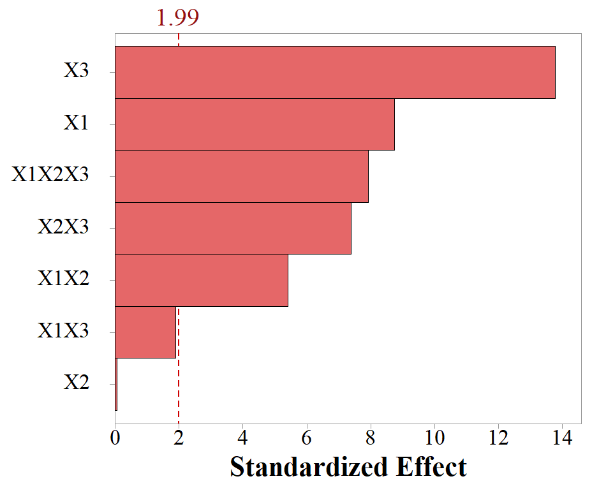** | **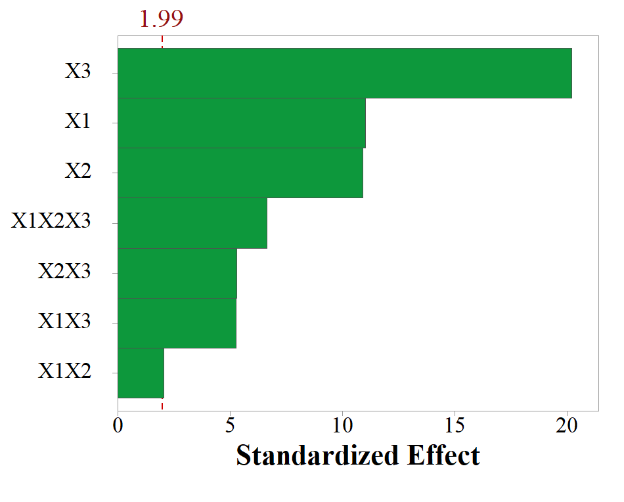** |
| **(C)** | **(D)** |
| **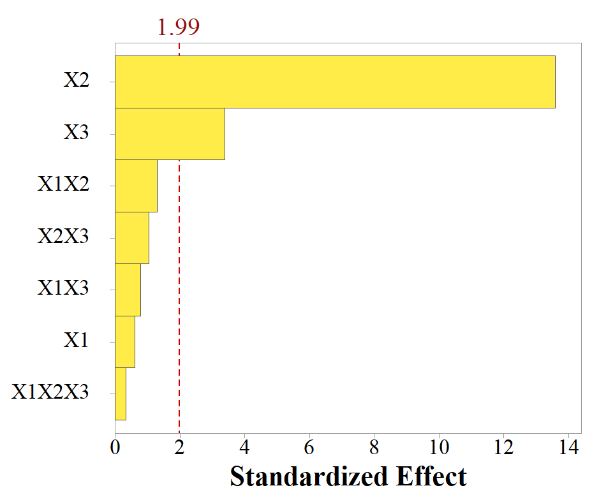** | **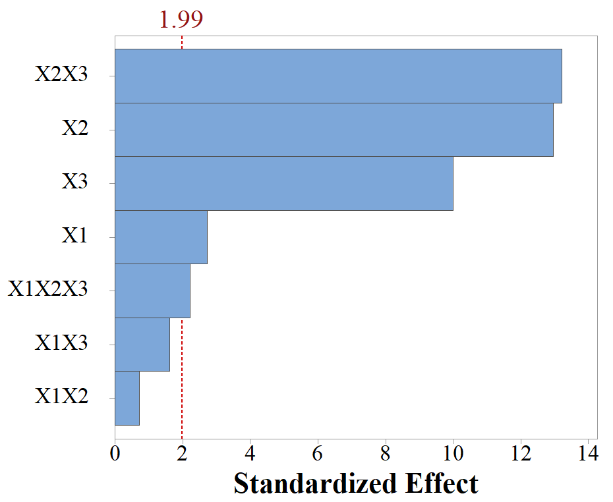** |

Figure S2. Pareto charts of (A) Porosity, (B) Swelling Ratio, (C) Degradation Rate (full model) and (D) Compressive Modulus, where X_1_ = t, X_2_ = HA% and X_3_ = Rib. The Pareto charts show the absolute values of the standardized effects from the largest to the smallest. The factors that cross the red line are considered statistically significant (level of significance α= 0.05 for each dataset).

| **(A)** | **(B)** |
| --- | --- |
| **** | **** |
| **(C)** | **(D)** |
| **** | **** |
| **Figure S3.** Residual Plots for the outputs **(A)** Porosity, **(B)** Swelling Ratio, **(C)** Degradation Rate (Reduced Model) and **(D)** Compressive Modulus.   \| **(A)** \| **(B)** \| **(C)** \| \| --- \| --- \| --- \| \| 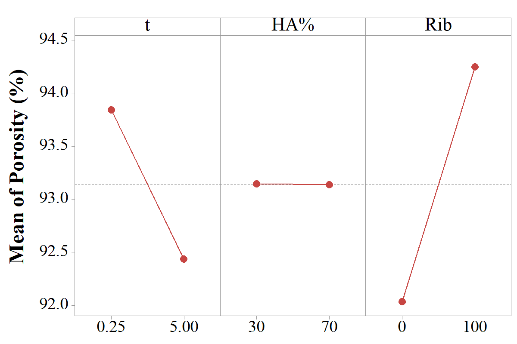 \| 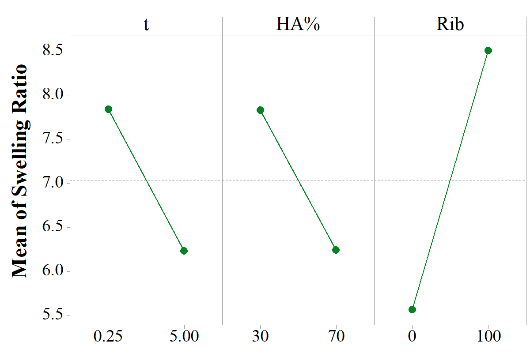 \| 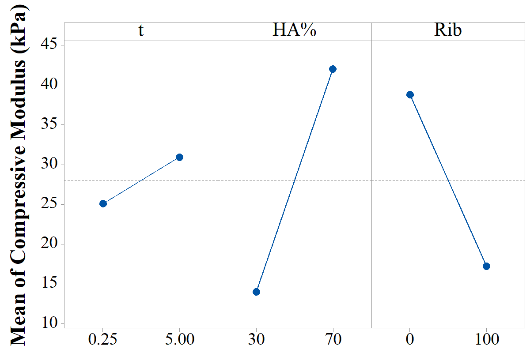 \| | |

**Figure S4**. Main effects plot for the outputs **(A)** Porosity, **(B)** Swelling Ratio and **(C)** Compressive Modulus.

**Figure S5.** Contour Plot of HA% versus Rib for Degradation Rate (Reduced Model). The parallelism of the isoresponse lines confirms the absence of relevant interactions between the variables.
